# Emerging Piezoelectric Metamaterials for Biomedical Applications

**DOI:** 10.53941/mi.2024.100004

**Published:** 2024-11-21

**Authors:** Zishuo Yan, Huy Tran, Dezun Ma, Jingwei Xie

**Affiliations:** 1 Department of Surgery-Transplant and Mary & Dick Holland Regenerative Medicine Program, University of Nebraska Medical Center, Omaha, NE 68198, USA; 2 Department of Mechanical and Materials Engineering, University of Nebraska Lincoln, Lincoln, NE 68588, USA

**Keywords:** piezoelectric metamaterials, molecular design, supramolecular packing, 3D assembly, biomedical applications

## Abstract

Emerging piezoelectric metamaterials hold immense promise for biomedical applications by merging the intrinsic electrical properties of piezoelectricity with the precise architecture of metamaterials. This review provides a comprehensive overview of various piezoelectric materials- such as molecular crystals, ceramics, and polymers—known for their exceptional piezoelectric performance and biocompatibility. We explore the advanced engineering approaches, including molecular design, supramolecular packing, and 3D assembly, which enable the customization of piezoelectric properties for targeted biomedical applications. Particular attention is given to the pivotal role of metamaterial structuring in the development of 0D spheres, 1D fibers and tubes, 2D films, and 3D scaffolds. Key biomedical applications, including tissue engineering, drug delivery, wound healing, and biosensing, are discussed through illustrative examples. Finally, the article addresses critical challenges and future directions, aiming to drive further innovations in piezoelectric biomaterials for next-generation healthcare technologies.

## 1. Introduction

As public focus and demand for health management continue to grow, the intersection of advanced materials science and biomedical engineering has given rise to a transformative field [1–3]. This development redefines the boundaries of medical science, aiming to address medical challenges and enhance patient care through innovative materials. One of the most promising engineered biomedical materials is piezoelectric metamaterials, which combine the electrically stimulating functional properties of piezoelectricity with the complex structural design possibilities of metamaterials [4,5]. Piezoelectric materials have the capacity to convert mechanical stress into electrical energy, a phenomenon arising from the intrinsic structural asymmetry of the material [6–8]. In piezoelectric materials, the non-centrosymmetric crystal structure induces the formation of electric dipoles. When mechanical stress is applied, the internal polarization of the material alters and results in a surface voltage. This capability has been utilized to stimulate cellular growth and repair in tissue regeneration, enhance controlled drug delivery through electrically regulated release mechanisms, accelerate wound healing by providing electrical stimulation to promote cellular migration and proliferation at the wound site and enable precise sensing of physiological and environmental changes by translating mechanical signals into electrical responses [9–13]. Piezoelectric materials are often oriented to enhance their performance and functionality, as the direction of their response is highly dependent on the alignment of their internal crystal structures [14–16].

Metamaterials, defined by their engineered structural properties, have evolved from their foundational applications in electromagnetism to a wide range of materials science fields, including energy harvesting, acoustic devices, telecommunications, and protective technologies [17–21]. A prominent feature of many metamaterials is their ordered structure, where repeating unit cells create a regular pattern. When combined with piezoelectric crystals, metamaterials offer even greater control over both electrical and mechanical properties [22–25]. Electrical charges generated by piezoelectric materials in response to mechanical stress can be precisely aligned within the metamaterial framework, optimizing their sensitivity and efficiency in converting mechanical energy into electrical energy [26–29]. The rapid development of engineered piezoelectric metamaterials is largely attributed to advancements in manufacturing techniques such as electrospinning, electrospray, 3D printing, and injection molding. These technologies enable precise manipulation of material properties and structures [30–36]. By integrating strategies such as interfacial induction, nanoconfinement, and high voltage in situ polarization, researchers can fabricate metamaterials with customized piezoelectric properties [37]. Furthermore, to be implemented in biological systems, the piezoelectric material must be endowed with properties like good mechanical flexibility, stretchability, high biocompatibility, and biodegradability [38]. Notable achievements include well-ordered 0D crystals, 1D nanofibers, 2D composite films, and 3D scaffolds, rendering them highly suitable for a wide range of implantable and wearable biomedical applications.

**Figure 1.**
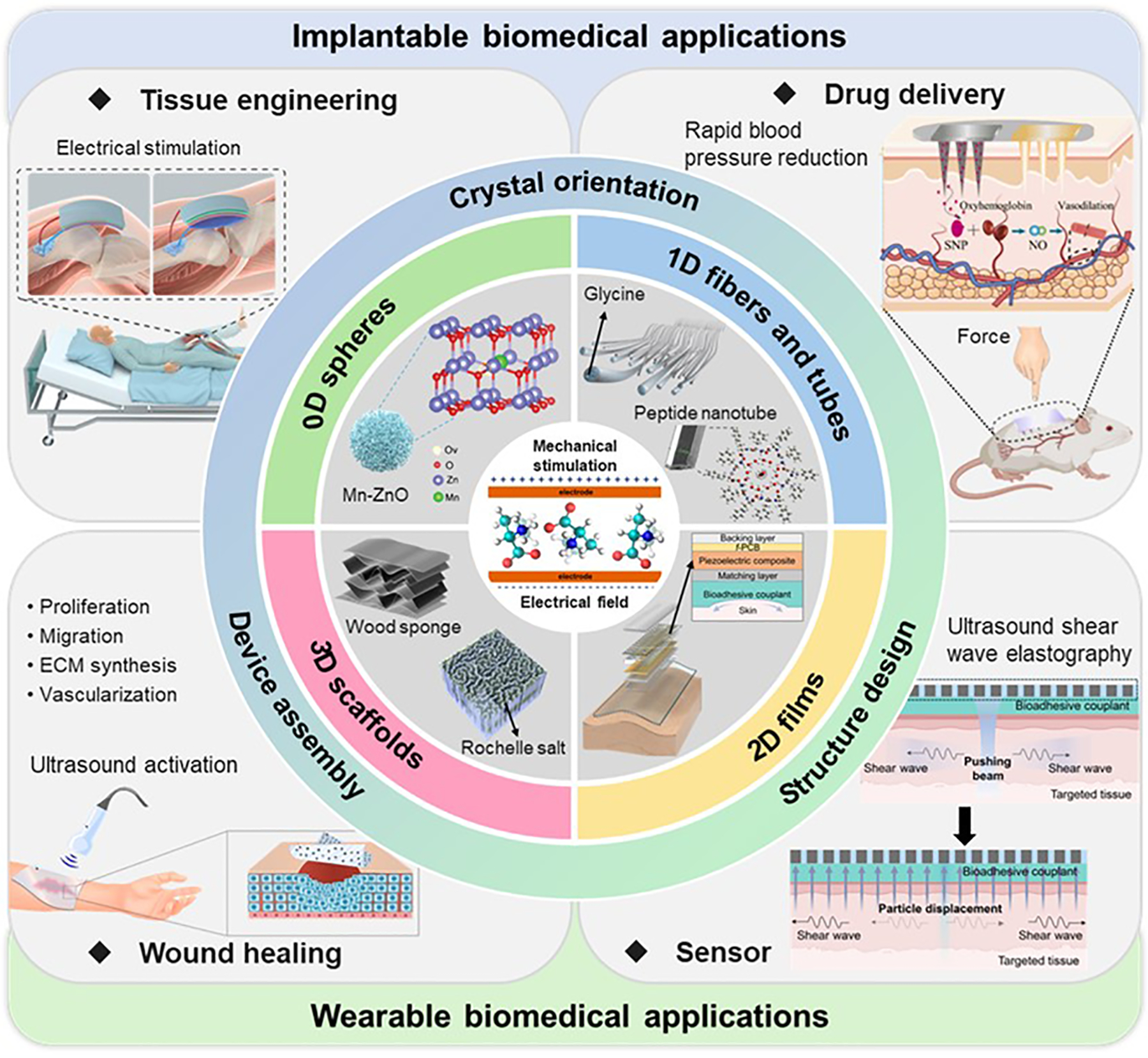
Schematic illustration of the overview of this work. Piezoelectric materials can be designed in different dimensional forms (e.g., 0D spheres, 1D fibers and tubes, 2D films, and 3D scaffolds) with their piezoelectric properties being tunable by controlling crystal orientation, structure design, and 3D assembly. This versatility allows for potential applications in biomedical fields including tissue engineering, drug delivery, wound healing, and biosensing. Tissue engineering: Reproduced with permission [39]. Copyright 2024, The American Association for the Advancement of Science. Drug delivery: Reproduced with permission [40]. Copyright 2024, Elsevier. Wound healing: Reproduced with permission [41]. Copyright 2023, Elsevier. Sensor and 2D films: Reproduced with permission [42]. Copyright 2024, The American Association for the Advancement of Science. 0D spheres: Reproduced with permission [43]. Copyright 2023, Wiley. 1D fibers: Reproduced with permission [44]. Copyright 2023, The American Association for the Advancement of Science. 1D tubes: Reproduced under the terms of the CC BY license [45]. Copyright 2019, Authors, published by AIP Publishing. Wood sponge: Reproduced with permission [46]. Copyright 2020, American Chemical Society. 3D scaffolds with Rochelle salt: Reproduced with permission [47]. Copyright 2023, Springer Nature. Piezoelectric effect: Reproduced with permission [48]. Copyright 2018, American Chemical Society.

In this review, we provide an overview of the latest advancements and innovations in piezoelectric biomedical materials, including types of materials, the construction of multi-level structures, and specific application examples (Figure 1). A brief outline of this review is as follows. Section 2 details the types of piezoelectric materials with exceptional piezoelectric activity and biocompatibility, including molecular crystals, ceramics, and polymers. The following section describes the methodologies for engineering these materials, introducing molecular design, supramolecular packing, and 3D assembly. Then, we present the performances and applications of piezoelectric materials in implantable and wearable biomedical fields, including tissue regeneration, drug delivery, wound healing, and biosensing. Finally, we discuss the challenges and future perspectives of piezoelectric biomaterials, aiming to provide a guide for further research and development to enhance the efficacy and application.

## 2. Piezoelectric Materials

To meet the stringent requirements of biomedical applications, piezoelectric materials should demonstrate both high piezoelectric coefficients and excellent biocompatibility. Since the piezoelectric effect arises in noncentrosymmetric crystals, this section provides a detailed overview of key piezoelectric materials, categorized into three major groups: (1) molecular crystals, (2) ceramics, and (3) polymers.

### 2.1. Molecular Crystals

**Figure 2.**
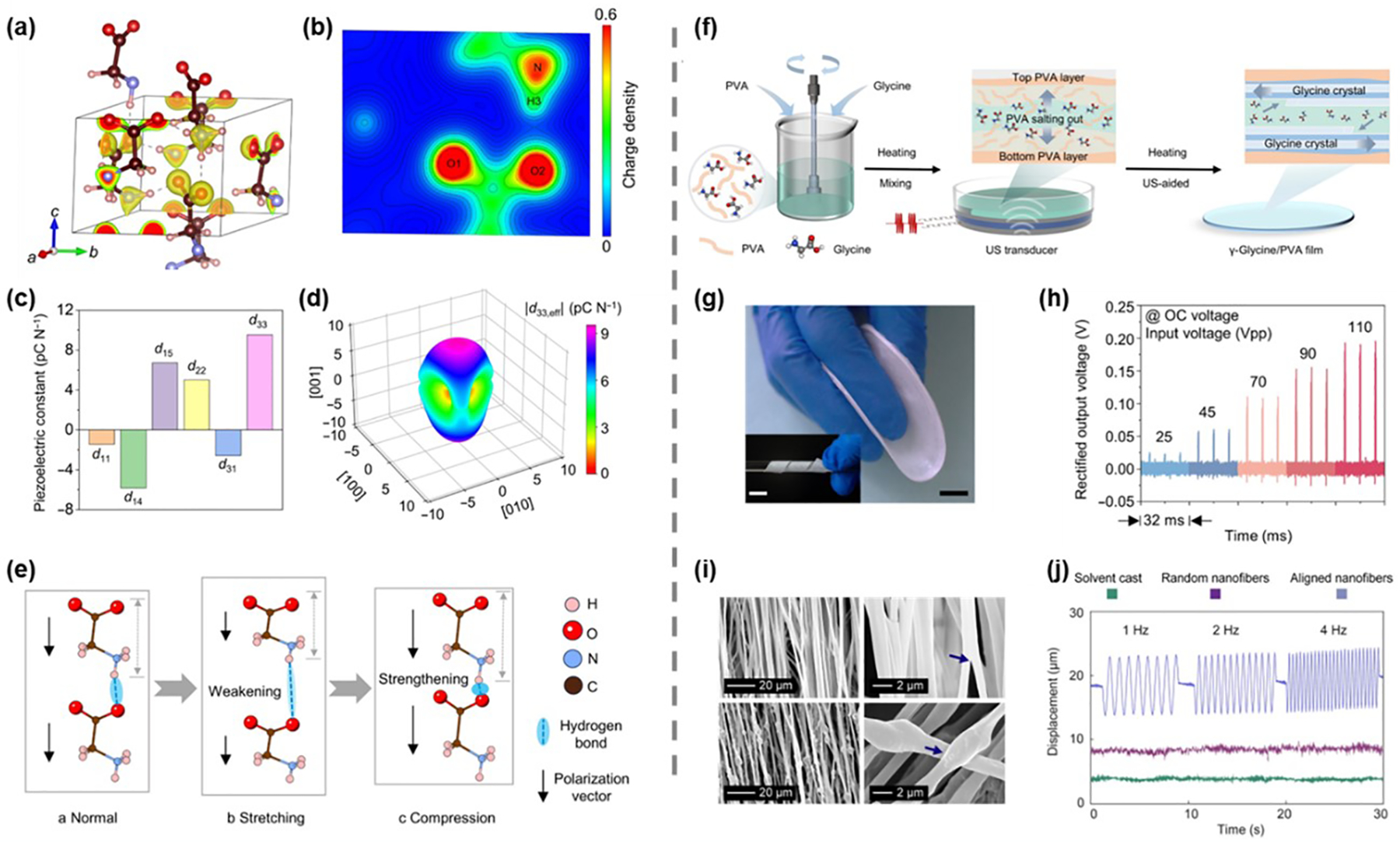
Glycine-based piezoelectric materials. (**a**) Schematic diagram and (**b**) contour plot of charge density distribution of γ-glycine molecules. (**c**) Piezoelectric constants of γ-glycine. (**d**) Orientation dependence of d_33,eff_. (**e**) The piezoelectric effect of γ-glycine induced by the hydrogen bonding interactions. (**f**) The fabrication process of γ-glycine/PVA films. (**g**) Optical photographs of the flexible γ-glycine/PVA films. The inset shows the twisted films. Scale bars, 10 mm. (**h**) Voltage output of glycine/PVA films. (**a**–**h**) Reproduced with permission [58]. Copyright 2024, The American Association for the Advancement of Science. (**i**) SEM images of PCL (top) and glycine-PCL (bottom) fibers. (**j**) Displacement of solvent-casting, random electrospinning, and aligned electrospinning films. (**i**–**j**) Reproduced with permission [44]. Copyright 2023, The American Association for the Advancement of Science.

Previous studies have demonstrated that various biological materials, both in the human body and nature, such as collagen, viruses, amino acids, cellulose, chitin, and silk, exhibit piezoelectric properties [49–54]. Among these, glycine, a simple and biocompatible amino acid, stands out due to its piezoelectricity in two of its crystalline forms: the β and γ-phases, while its α-phase is non-piezoelectric [55]. Yang et al. employed electrohydrodynamic spraying to fabricate piezoelectric β-glycine films, which resemble the polycrystalline morphology found in inorganic materials [56]. Their approach combined nanoconfinement and in situ electric fields during glycine nucleation and self-assembly, simulating the sintering and polarization steps used piezoelectric ceramic fabrication. Similarly, Wang et al. prepared wafer-scale heterostructured piezoelectric glycine films using a direct solidification process [57]. The hydrogen bonding between glycine and polyvinyl alcohol (PVA) at the interface facilitated the formation of self-aligned γ-glycine crystals with enhanced piezoelectricity. Building on this, Wu et al. focused on optimizing glycine’s piezoelectric potential by refining its crystal orientation [58]. Their study, employing density functional theory (DFT), examined γ-glycine’s crystal structure and the origin of its piezoelectric properties. Figure 2a,b depict the charge density distribution, highlighting the high charge densities of O and N atoms, with N atoms extending to H3, suggesting the formation of hydrogen bonds with surrounding molecules. Among the piezoelectric coefficients, the d_33_ was identified as the most significant, with a value of 9.53 pC N^−1^ (Figure 2c). Further analysis revealed that optimal piezoelectric performance of γ-glycine is achieved when the crystals are oriented at the smallest possible angle on the substrate (Figure 2d). Additionally, when glycine crystals are stretched, the increased intermolecular distance weakens hydrogen bonds, causing molecular contraction and a corresponding reduction in polarization (Figure 2e). This highlights the sensitivity of glycine’s piezoelectric properties to molecular interactions and mechanical strain.

To advance the development and application of γ-glycine in biomedical applications, self-assembled γ-glycine/PVA films were synthesized using an ultrasonic-assisted mixing-solidification technique (Figure 2f,g). The output voltages of these films increased proportionally with trigger voltages, reaching approximately 220 mV at 110 Vpp (Figure 2h). In addition to direct solidification, Nguyen et al. used electrospinning to fabricate glycine-poly(e-caprolactone) (PCL) nanofibers with highly oriented glycine crystals [44]. This hybrid electrospinning technique, which involves high voltage and rapid collector speeds, stretches and aligns the nanofibers, resulting in enhanced piezoelectric properties in glycine-PCL nanofibrous membranes [59]. The applied electric field during electrospinning improves the polarization of glycine within the fibers by reorienting the crystalline dipoles. Scanning electron microscopy (SEM) images of PCL and glycine-PCL nanofibers revealed their surface morphology and the incorporation of glycine crystals within individual nanofibers (Figure 2i). The aligned electrospun membranes demonstrated a displacement of approximately 12 μm, in contrast to solvent-cast and randomly oriented samples, which showed no measurable displacement (Figure 2j). These results suggest that aligned glycine-PCL nanofibrous membranes hold significant potential as actuators for ultrasonic applications under applied voltage. However, most glycine-based materials are currently limited to out-of-plane piezoelectric responses, which correspond to longitudinal and transverse piezoelectric coefficients. This leaves the full potential of shear piezoelectricity yet to be explored. Notably, β-glycine exhibits piezoelectric responses as high as 180 pC N^−1^ [60]. Future research should focus on optimizing the spatial arrangement of these materials to create stress distributions dominated by shear forces, enabling more effective utilization of shear piezoelectricity in integrated devices.

### 2.2. Ceramics

**Figure 3.**
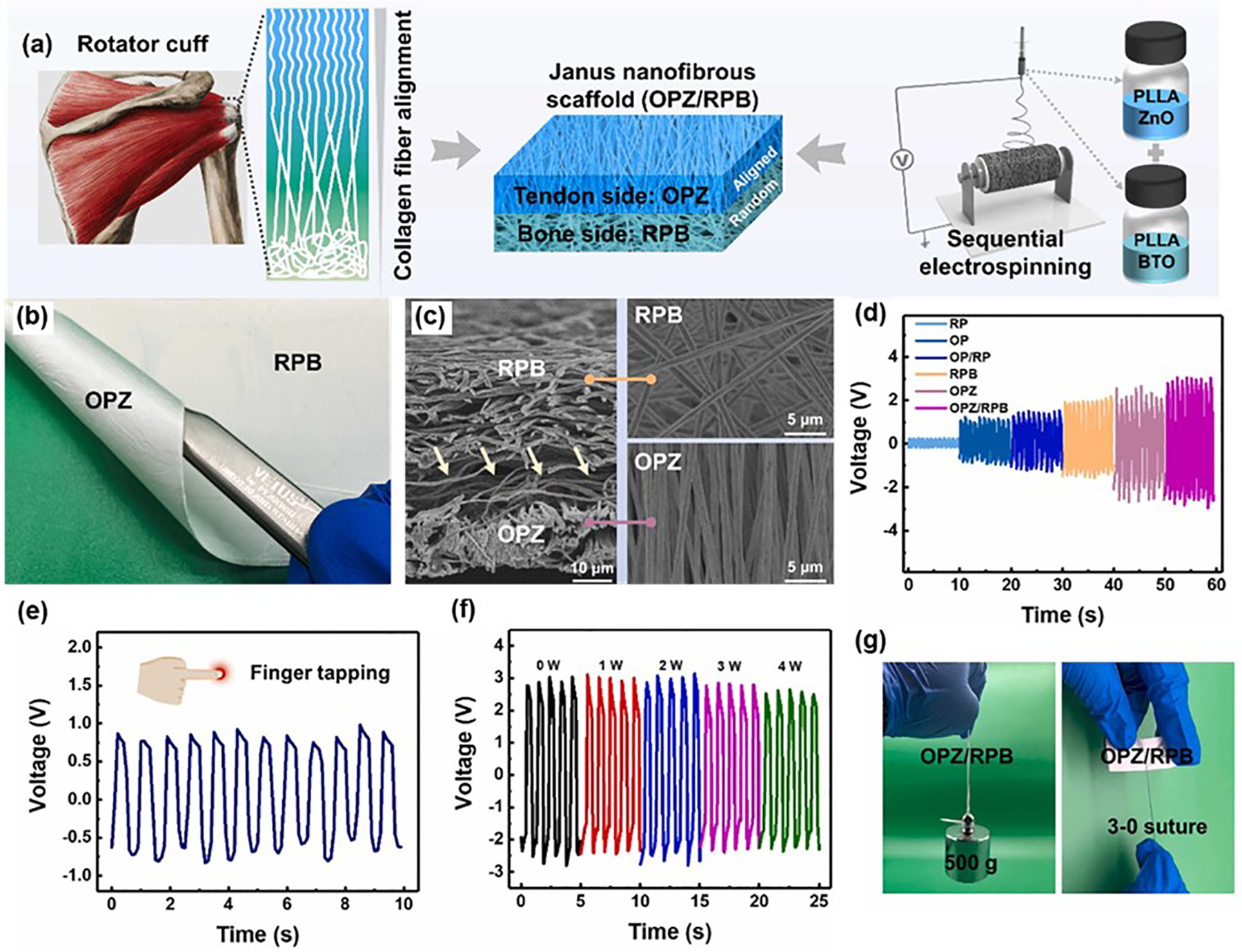
Ceramic-based piezoelectric materials. (**a**) The fabrication process of the Janus piezoelectric nanofibrous scaffolds. (**b**) The optical photographs of the OPZ/RPB scaffold. (**c**) The SEM images of the OPZ/RPB scaffold. (**d**) Voltage output of the scaffolds under an external periodic impact force (20 kPa, 1 Hz). (**e**) Voltage output of the OPZ/RPB scaffold under slight finger tapping. (**f**) The maintenance of the piezoelectric output during the degradation period. (**g**) The mechanical performances of the OPZ/RPB scaffold. Reproduced with permission [70]. Copyright 2024, Elsevier.

Some of the earliest piezoelectric materials identified, such as quartz and Rochelle salt, were utilized in ultrasound technologies throughout the 20th century [61,62]. In recent years, ceramics with excellent piezoelectric properties and chemical stability, such as biocompatible zinc oxide (ZnO) and zinc stannate, have been applied in biomedical applications [63–65]. Cauda et al. investigated the effectiveness of piezoelectric ZnO micro- and nanoparticles in ultrasound-assisted sonodynamic therapy for cancer treatment [66]. Their research demonstrated that the combined ZnO-ultrasound treatment exhibited remarkable efficacy against osteosarcoma and glioblastoma cell lines in vitro. ZnO’s role as a catalyst for reactive oxygen species (ROS) production under ultrasound underscores its therapeutic potential. However, the intrinsic rigidity and brittleness of ceramic materials limit their mechanical tolerance to defects and external strains [67–69]. To address these limitations, bio-piezoelectric ceramics have been incorporated into flexible films and fibers to enhance mechanical robustness. For example, Zhu et al. developed a Janus nanofibrous scaffold with piezoelectric properties designed to promote tendon-to-bone healing [70]. The scaffold was prepared by electrospinning poly (_L_-lactic acid) (PLLA)/ZnO (OPZ) and PLLA/barium titanate (RPB), with fiber alignment varying between the layers by manipulating the rotating speed of the roller (Figure 3a). The OPZ layer featured aligned fibers, while the RPB layer contained randomly oriented fibers, mimicking the natural structural of collagen fibers in tendons and bones. This specific configuration was aimed at promoting topographical effects that support tendon-to-bone healing. The OPZ/RPB scaffold possessed remarkable structural integrity and flexibility. The OPZ layer’s fibers had an average diameter of 756.84 nm, while the RPB layer’s fibers measured 986.52 nm in diameter (Figure 3b,c). As illustrated in Figure 3d, the scaffold’s piezoelectric performance significantly outperformed pure PLLA scaffolds, with a 110.78% increase in voltage output compared to a bilayered oriented PLLA/random PLLA (OP/RP) scaffold. Furthermore, the OPZ/RPB scaffold was able to generate real-time electrical signals in response to subtle body movements (Figure 3e). The durability of the biocompatible scaffold was evidenced by maintaining over 80% of its piezoelectric output even after four weeks of immersion in a PBS solution, highlighting its exceptional capacity for providing electrical stimulation (Figure 3f). In addition, the OPZ/RPB scaffold has outstanding mechanical properties, making it highly suitable for rotator cuff repair (Figure 3g). While combining ceramic crystals with polymer matrices in flexible composites enhances performances, it is crucial to address a commonly overlooked issue the viscoelastic properties of the polymer matrix. Over time, polymer creep can occur under load, reducing stress transfer efficiency and ultimately affecting the piezoelectric performance of the device. This issue must be a focus of future research and development to further optimize the long-term functionality of these flexible piezoelectric composites.

### 2.3. Polymers

**Figure 4.**
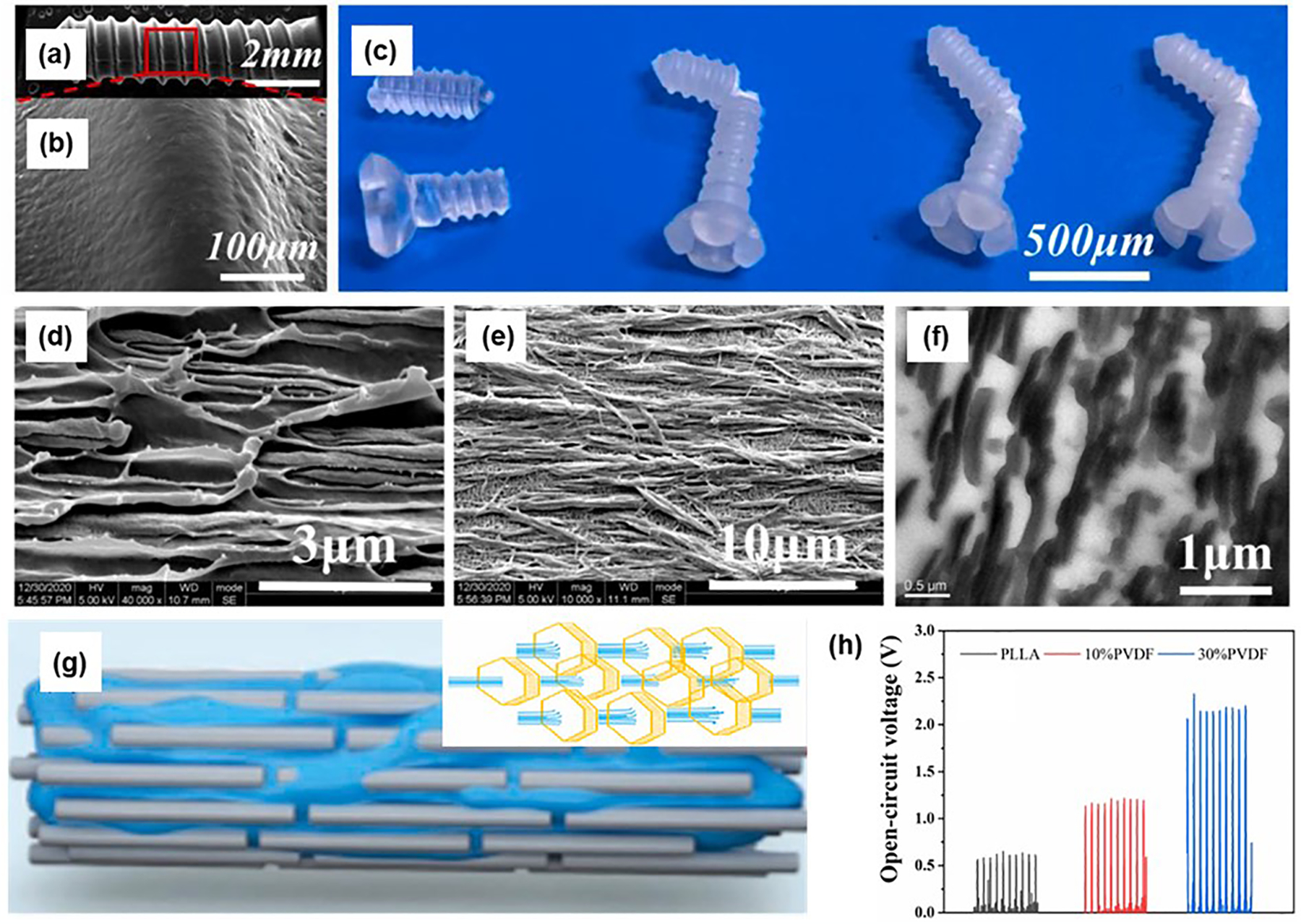
Polymer-based piezoelectric materials. (**a**,**b**) The SEM images of micro bone screw. (**c**) Optical photographs of the micro bone screw after three-point bending test (from left to right: 0 wt%, 10 wt%, 20 wt%, and 30 wt% PVDF, respectively). The morphology characterization of PVDF dispersed phase fibers: (**d**) SEM images of cryo-fracture surface, (**e**) after etching PLLA matrix and (**f**) transmission electron microscopy image. (**g**) The formation of the submicron PVDF fibers. (**h**) The piezoelectric performances of micro bone screws. Reproduced with permission [75]. Copyright 2024, Elsevier.

Polymers with asymmetrical molecular structures and orientations are highly attractive as piezoelectric materials due to their superior mechanical flexibility, ease of low-temperature processing, and design versatility compared to inorganic alternatives. In particular, biodegradable and biocompatible polymers like PLLA and its composites are widely employed in implantable and wearable biomedical applications [71–73]. Wang et al. developed a core-shell structure of PLLA/glycine nanofibers using an electrospinning technique through an interfacial anchoring strategy [74]. These nanofibers exhibit a high proportion of piezoelectric β-phase and excellent alignment. The self-assembled core-shell architecture promoted strong intermolecular interactions between the hydroxyl groups of glycine and the carbonyl groups of PLLA, facilitating the formation and alignment of β-phase in PLLA. Piezoresponse force microscopy (PFM) which was used to characterize the piezoelectric properties revealed a *d*^eff^33 value of 2.21 pm V^−1^ for individual fibers. A stable voltage output with a peak-to-peak voltage of 1.2 V is achieved under repetitive pressure applied over a vertical distance of 8 mm at a frequency of 4 Hz. In addition to nanofibers, Tang et al. utilized microinjection molding to fabricate hybrid PLLA/polyvinylidene fluoride (PLLA/PVDF) micro bone screws with enhanced toughness for bone repair [75]. SEM images of the screws showed high-quality fabrication with smooth surfaces and no visible defects (Figure 4a,b). After bending tests, digital images confirmed the improved mechanical properties of the screws, attributed to the incorporation of PVDF (Figure 4c). During microinjection molding, the shear stress field induced the in-situ formation of highly oriented PVDF fiber arrays (Figure 4d–g). These aligned PVDF fibers significantly enhanced both the toughness and strength of the micro bone screws. Piezoelectric testing demonstrated that these screws could generate an open-circuit voltage of up to 2 V (Figure 4h). Although multiple physical field methods including electric field and directional shear force field could induce phase transitions or orientation alignment in polymer crystals, the piezoelectric performance of polymer-based materials remains significantly lower than that of piezoelectric ceramics and single crystals. To meet higher performance demands, future research should focus on constructing porous structures to enhance polarization efficiency and improve the piezoelectric properties of polymer-based materials.

Table 1 summarizes the piezoelectric properties of various biomaterials, including their composition, fabrication methods, structures, piezoelectric performances, and applications. In brief, piezoelectric ceramic materials are renowned for their high piezoelectric performance, making them valuable for a wide range of applications. However, their inherent rigidity and brittleness limit their integration into flexible or dynamic systems. Furthermore, many piezoelectric ceramics lack biodegradability and biocompatibility, necessitating restrictions of their use in medical devices. In contrast, piezoelectric polymers are flexible, lightweight, and easily deployable. However, repeated mechanical stress and strain could induce changes in their internal structures through mechanisms such as creep. The accumulation of such changes ultimately leads to a gradual decline in their piezoelectric performance of the polymers. Molecular crystals, such as amino acids, exhibit excellent biocompatibility and biodegradability. Despite these advantages, their piezoelectric performance is relatively low, often relegating them to roles as fillers in polymer matrices.

## 3. Multi-Level Structural Construction Strategies

Recent advancements in mechanical engineering and material fabrication technologies have paved the way for high-performance piezoelectric devices, unlocking new possibilities for biomedical applications. This section highlights key strategies to enhance piezoelectric performance, while also improving structural stability and durability through innovations in molecular design, supramolecular packing, and 3D assembly techniques.

### 3.1. Molecular Design

**Figure 5.**
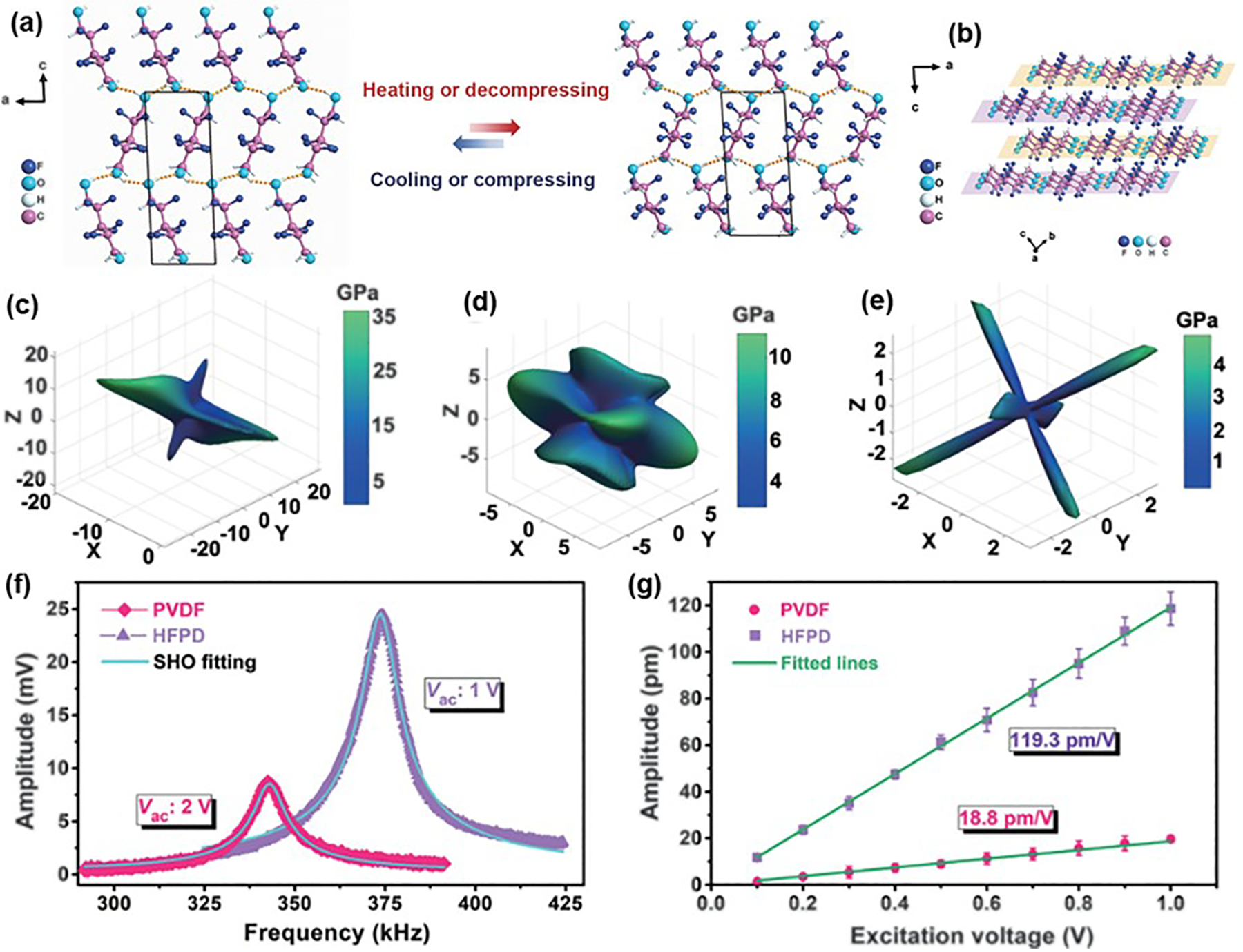
Molecular design for enhancing piezoelectric properties. (**a**) The transition of 2D monolayer formed through O−H⋯O interactions. (**b**) Packing view of 2D hydrogen bond layers. 3D plots of elastic modulus of HFPD crystals: (**c**) Young’s modulus, (**d**) shear modulus MAX, and (**e**) shear modulus MIN. (**f**) Piezoelectric response of HFPD and PVDF films versus excitation frequency measured with PFM. (**g**) The derived amplitude curve for HFPD and PVDF films. Reproduced with permission [87]. Copyright 2024, The American Association for the Advancement of Science.

Although biodegradable materials such as collagen, cellulose, and PLLA have been investigated for biomedical applications, there remains a need for biomaterials with superior piezoelectric properties [22]. In this context, Xiong et al. pioneered the development of a molecular crystal HOCH_2_(CF_2_)_3_CH_2_OH [2,2,3,3,4,4-hexafluoropentane-1,5-diol (HFPD)], exhibiting a remarkable piezoelectric response of 138 pC N^−1^, which is more than 13 times higher than glycine [87,88]. HFPD molecules form a 2D hydrogen-bonded layer through O−H⋯O interactions, while the asymmetric distribution of C-F bonds creates non-zero molecular dipole moments, aligned by the hydrogen-bonded structure (Figure 5a,b). The mechanical properties of HFPD crystals revealed significant anisotropy in the directional Young’s modulus, with two maximum values aligned on an elliptical plane. This anisotropy stems from the 2D hydrogen-bonding network and the ordered arrangement of F atoms (Figure 5c). The spatial dependence of the shear modulus also displays a shell-like structure, where the MIN surface is nested within the MAX surface, contributing to the high piezoelectric coefficient (Figure 5d,e). The piezoelectric performance of HFPD was characterized using PFM (Figure 5f,g), showing a much higher response peak compared to PVDF. The estimated piezoelectric coefficient for HFPD is 119.3 pm/V, significantly outperforming PVDF’s 18.8 pm/V. These findings suggest that HFPD molecular crystals could be developed into flexible piezoelectric films suitable for drug delivery, energy-harvesting devices, and tissue engineering scaffolds. In parallel, Ji et al. investigated how molecular design could enhance the piezoelectricity of amino acid-based materials [89]. They found that chemically modifying amino acid side chains through acetylation could increase molecular polarization, significantly enhancing the piezoelectric response. For example, acetylated tryptophan was predicted to have a piezoelectric strain constant of 47 pm V^−1^, comparable to that of bismuth triboride. These results emphasize the critical role of molecular design in optimizing the piezoelectric properties of biomolecular crystals.

### 3.2. Supramolecular Packing

**Figure 6.**
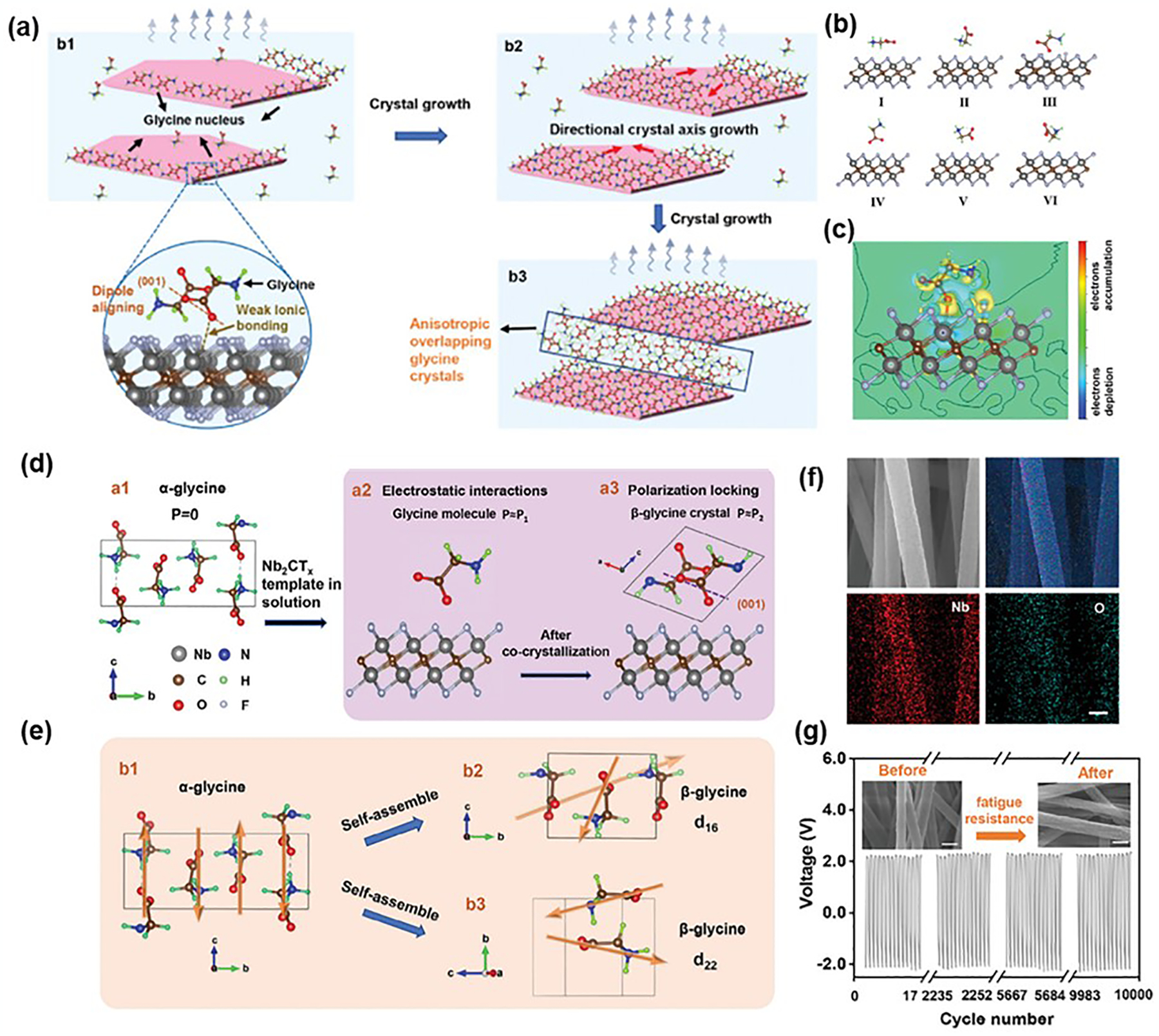
Supramolecular packing for enhancing piezoelectric properties. (**a**) The growth mechanism of glycine on Nb_2_CT_x_ nanosheets. (**b**) Adsorption configurations of glycine-Nb_2_CT_x_ nanosheets. (**c**) Difference of charge density contour on adsorption plane. (**d**) Formation of piezoelectric glycine induced by Nb2CTx nanosheets. (**e**) Piezoelectric response in glycine crystals with molecular dipoles. (**f**) SEM image of obtained nanofibers and corresponding EDS map of O and Nb elements. Scale bar = 400 nm. (**g**) Stability of the piezoelectric output. The inset shows the SEM image before and after fatigue resistance. Scale bar = 400 nm. Reproduced with permission [93]. Copyright 2024, Wiley.

Supramolecular packing involves the organized assembly of molecules through non-covalent interactions, such as hydrogen bonding, van der Waals forces, and π-π stacking. Researchers have employed various strategies, including template-assisted methods, dynamic self-assembly, and the use of external electrostatic and magnetic fields, to align molecules along specific crystallographic planes and directions, thereby affecting the overall dipole moment and enhancing piezoelectric properties [90–92]. For instance, Thompson et al. demonstrated how co-crystallization can enhance the piezoelectric properties of materials by using glycine and sulfamic acid as examples [60]. While these co-formers typically crystallize in centrosymmetric space groups when grown independently, co-crystallization leads to the formation of non-centrosymmetric, piezoelectrically active ionic co-crystals. In a related study, Feng et al. engineered flexible β-glycine/Nb_2_CT_x_ piezoelectric nanofibers using a nanoconfinement self-assembly approach, which locked aligned crystal domains through interfacial polarization (Figure 6a) [93]. Nb_2_CT_x_ nanosheets acted as nucleating agents, guiding the crystallization of glycine from the edges to the surfaces along its 2D crystal plane. This spontaneous kinetic crystallization process allowed precise control over the orientation of the glycine crystals. To elucidate the mechanism of the polarization templating, the interaction between glycine and the nanosheets was explored through DFT (Figure 6b,c). The alignment orientations and the electronic charge density difference map indicate the formation of weak ionic bonds between glycine and Nb_2_CT_x_ nanosheets, facilitating preferential crystallization of glycine on the nanosheet surfaces. After the co-crystallization process, glycine crystallizes into β-phase, optimally oriented on the Nb_2_CT_x_ nanosheets, forming an interfacial polarization lock. This configuration maintained a non-zero net polarization in the glycine crystals, yielding piezoelectric coefficients d_16_ and d_22_ (Figure 6d,e). Experimental results demonstrated that these glycine/Nb_2_CT_x_ co-crystals grew uniformly on aligned nanofiber substrates, generating a stable output voltage of 4.3 V under a stress of 10 N (Figure 6f,g).

### 3.3. Three-Dimensional Assembly

**Figure 7.**
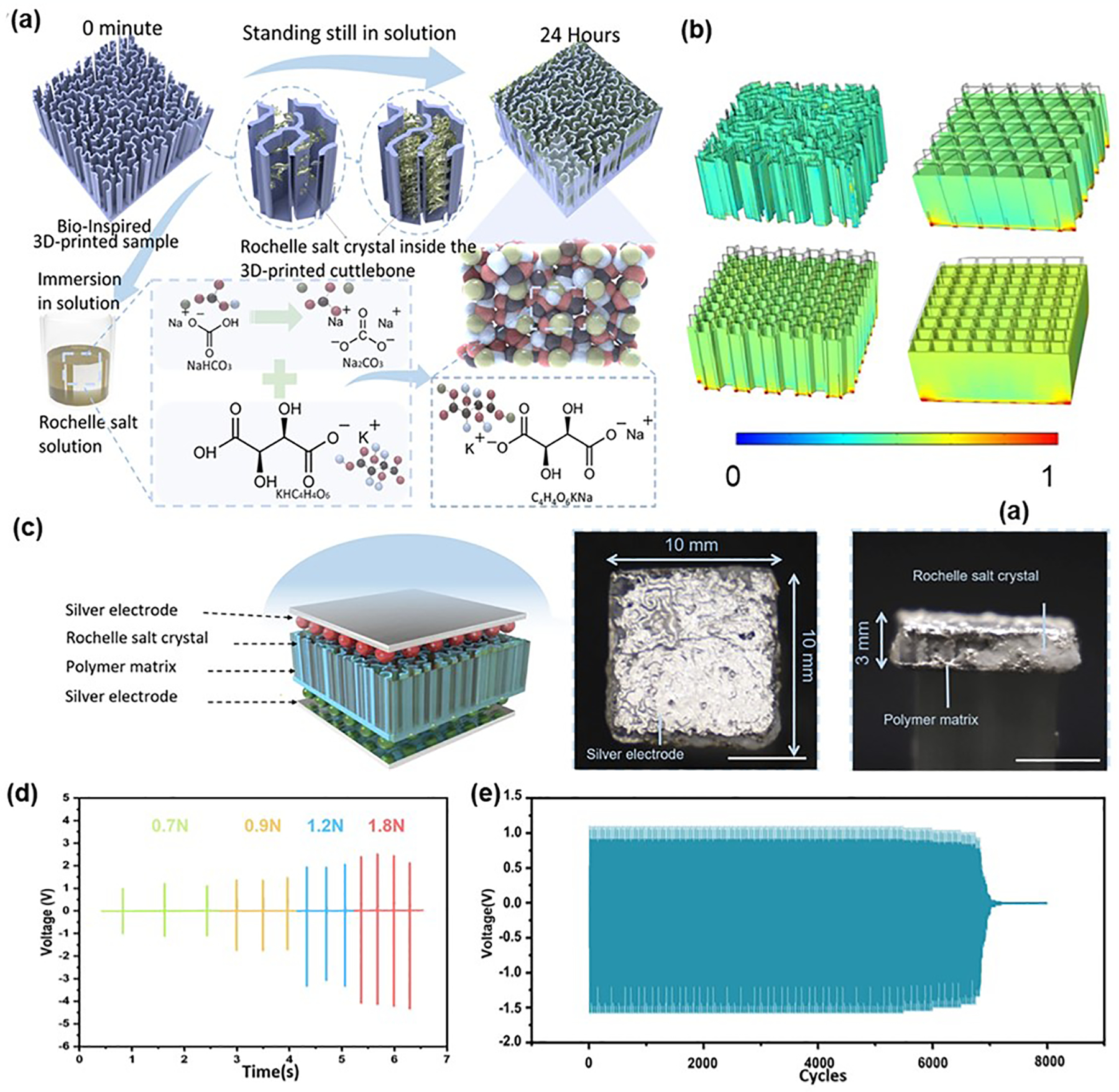
3D assembly for enhancing piezoelectric properties. (**a**) Fabrication process of bio-inspired 3D-printed cuttlefish bone structure and Rochelle salt crystal. (**b**) Simulation results of the stress distribution under compressive loading. (**c**) Schematic of 3D printed sample for piezoelectric performance testing. Scale bar = 5 mm. (**d**) Voltage output at different frequencies. (**e**) Voltage output over 8000 cycles cyclic impact test (2 Hz). Reproduced with permission [47]. Copyright 2023, Springer Nature.

3D piezoelectric materials, known for their complex structures, superior mechanical properties, lightweight nature, and enhanced surface activity, hold significant promise for applications in biomedical sensors, tissue engineering scaffolds, and wearable devices [94–97]. Inspired by the unique chambered wall-septa microstructure of cuttlebone, Yang et al. developed a biomimetic 3D piezoelectric composite with enhanced mechanical and electrical properties [47]. They utilized stereolithography to create a photocurable resin model mimicking the cuttlebone structure, then grew recyclable piezoelectric Rochelle salt crystals within the resin framework (Figure 7a). When compared to other conventional structures with the same dimensions (e.g., cubic, honeycomb, and triangular), the cuttlebone-inspired composite exhibited the least deformation and most uniform stress distribution under compressive loads (Figure 7b). As shown in Figure 7c, the composite was integrated into devices with silver electrodes to evaluate its piezoelectric properties, showing a robust response to varying force levels and producing significant voltage outputs. Moreover, the composite exhibited excellent durability, maintaining stable voltage output over 6800 shock cycles (Figure 7d,e). These biomimetic 3D piezoelectric composites offer both exceptional mechanical protection and impressive sensing capabilities, making them ideal for applications such as smart knee pads. These pads can deliver integrated mechanical protection, act as fall detection alarms, and collect data for medical evaluation. In addition to biomimetic design, advanced technologies such as 3D printing and 3D weaving have been employed to assemble complex piezoelectric materials [98,99]. These fabrication methods enable the creation of complex, customized structures that enhance the functionality and performance of piezoelectric composites, paving the way for innovative applications and improved integration into various smart devices.

## 4. Biomedical Applications

The electromechanical conversion capabilities of piezoelectric bio-systems allow them to convert external stimuli into electrical energy, making them ideal for a wide range of biomedical applications. In recent years, significant advancements have been made in both implantable and wearable devices leveraging this technology [100–104]. Research has primary focused on four key areas: (i) Stimulating cellular growth and tissue repair and regeneration; (ii) Enhancing drug delivery by improving molecular release and permeability; (iii) Directing and accelerating cell migration to promote wound healing; and (iv) Capturing multimodal physiological signals for non-invasive diagnostic purposes.

### 4.1. Tissue Regeneration

Over the past decades, piezoelectric biomaterials have shown great potential in tissue healing and the restoration of cellular functions. Biomedical engineers have used dynamic electric stimulation on these materials to activate, proliferate, and differentiate cells, aiding the repair of various tissues including nerves, muscles, skin, and bone [105–108]. For example, Wang et al. fabricated electrospun fibers composed of PCL embedded with piezoelectric tetragonal-SrTiO_3_ to mimic the native tendon structure for tendon injury repair [109]. The tetragonal-SrTiO_3_ enhanced the expression of tendon-related genes, and promoted collagen deposition, and reduced inflammation. Similarly, Liu et al. created a conductive piezoelectric BaTiO_3_/perampanel silk fibroin hydrogel capable of wireless electrical stimulation triggered by ultrasound [110]. This hydrogel not only showed excellent electrical conductivity but also anti-glutamate excitotoxicity, enhanced motor function recovery, and promoted spinal cord regeneration in a spinal cord injury rat model. During bone regeneration, electrical stimulation enhances Ca^2+^ influx by activating voltage-gated calcium channels (VGCCs) and triggering the Ca^2+^ signaling pathway, thereby promoting osteogenic differentiation [111,112]. In nerve regeneration, an increase in intracellular calcium concentration can induce actin depolymerization and contraction of this side of the cell, directing the cell’s movement toward the cathode [113,114]. Similarly, in neural stem cell differentiation, applying an electric field modulates calcium signaling at the early stage, effectively steering differentiation toward neuronal lineages [115].

#### 4.1.1. Bone Regeneration

**Figure 8.**
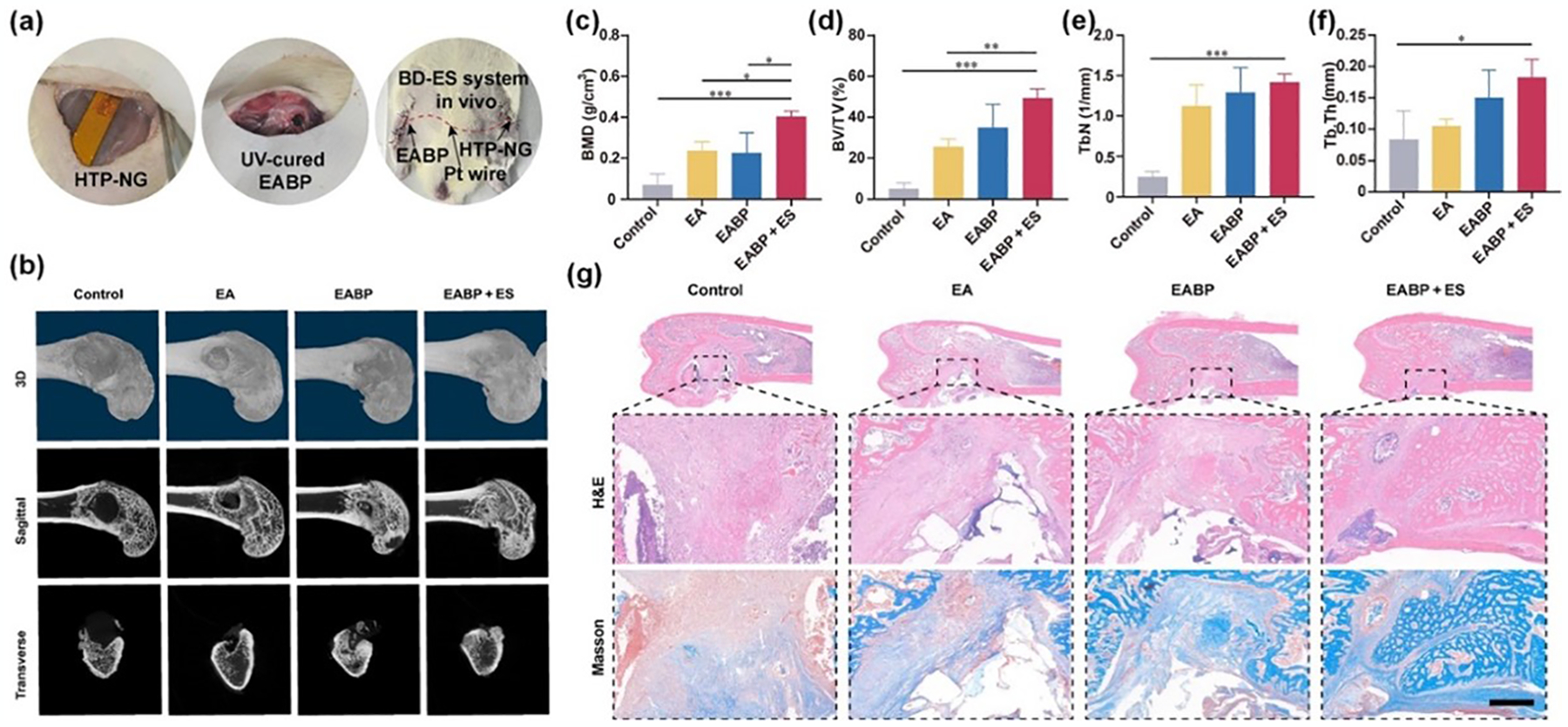
Implantable electrical stimulation device made of tribo- and piezoelectric nanogenerators for enhancing bone regeneration. (**a**) Surgical images of the implanted system. (**b**) 3D reconstruction images and sagittal and transverse view images of the distal femur by micro-CT. (**c**–**f**) Micro-CT quantitative evaluation of BMD, BV/TV, Tb. n, and Tb. Th in defect areas. Data are expressed as means ± SD. One-way ANOVA with Tukey’s multiple comparisons test, *** *p* < 0.001 and * *p* < 0.05, n = 3. (**g**) H&E and Masson’s trichrome staining images of the rat femurs. Scale bar = 500 μm. Reproduced with permission [39]. Copyright 2024, The American Association for the Advancement of Science.

Zheng et al. developed a fully implantable bone defect electrical stimulation (BD-ES) device, which integrates a hybrid tribo-/piezoelectric nanogenerator (HTP-NG) to generate electric pulses in response to rehabilitation movements [39]. This system was coupled with a conductive bioactive hydrogel made from ECM/alginate methacryloyl/polydopamine modified black phosphorus nanosheets (EABP) (Figure 8a). The BD-ES system facilitated the creation of an osteogenic microenvironment at the defect site by triggering biological processes, including calcium ion influx, cell proliferation and migration, and osteogenic differentiation. The piezoelectric materials used in the device consisted of commercial poled PVDF films with silver electrodes on both sides. In vivo studies on the osteogenic properties of the BD- ES system involved placing the HTP-NG near the knee joint and drilling a 3-mm-diameter cylindrical defect in the medial femur condyle. The HTP-NG was connected to the defect site via subcutaneously implanted platinum wires. The device efficiently harvested energy from rat knee joint’s movements, generating electric pulses. Micro-CT scans demonstrated that the group treated with EABP and ES showed enhanced osteogenic induction compared to the control groups (ECM/alginate methacryloyl hydrogel (EA) and EABP without stimulation) (Figure 8b). Morphological parameters such as bone mineral density (BMD), bone volume (BV)/total volume (TV), trabecular number (Tb. N), and trabecular thickness (Tb. Th) were also assessed, with the EABP + ES group exhibiting significantly improved bone regeneration (Figure 8c). The BV/TV ratio was 1.4 times greater in the EABP + ES group than in the EABP group (Figure 8d), and Tb. N and Tb also showed significant differences (Figure 8e,f). Additionally, histological analyses using hematoxylin and eosin (H&E) and Masson trichrome staining demonstrated a marked increase in newly formed bone and collagen in the EABP + ES group (Figure 8g). The BD-ES system demonstrates the potential for integrating rehabilitation exercise with electrical stimulation to enhance bone regeneration, offering new insights for the design of self-responsive devices for intelligent healthcare. This innovation holds promise for improving the treatment of clinical BD.

#### 4.1.2. Wound Healing

**Figure 9.**
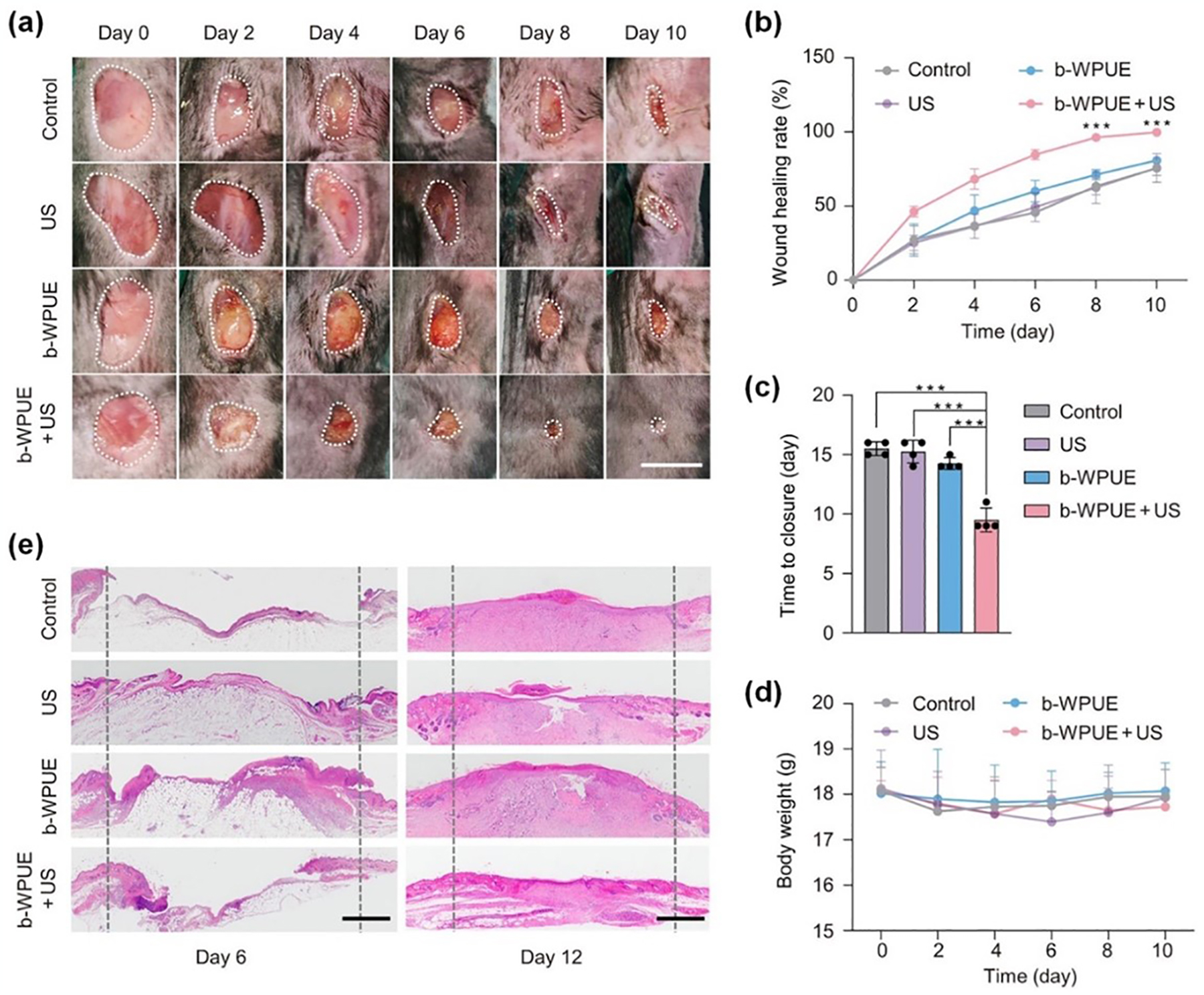
Bioresorbable ultrasonic wireless electrotherapy device made of piezoelectric γ-glycine/PVA biofilm for enhancing wound healing. (**a**) Optical photographs of wound healing in mice with different treatments. Scale bar = 6 mm. (**b**) Quantitative analysis of wound healing from 0 to 10 days (n = 4; ***p < 0.001). (**c**) Summary of the complete wound closure times (n = 4; *** *p* < 0.001). (**d**) Mice weight changes during wound treatments (n = 4). (**e**) H&E staining images of wound sections at days 6 and 12 after wounding. Scale bar = 1 mm. Reproduced with permission [58]. Copyright 2024, The American Association for the Advancement of Science.

Wound healing is crucial for maintaining the integrity of multicellular organisms. Numerous studies have demonstrated that disruption of the epithelial layer generates endogenous electric fields (EF) that play a key role in directing and accelerating cell migration [116,117]. However, the clinical application of exogenous EF to promote wound healing is often hindered by the need for cumbersome and complex ES devices at the injury site. To address this challenge, Sun et al. developed a flexible sono-piezo patch comprised of multifunctional fibers embedded with piezoelectric ZnO nanoparticles [118]. This patch leveraged low-intensity pulsed ultrasound to activate ES in the target tissues, which enhanced pro-regenerative behaviors in surrounding tissues and cells. Similarly, bio-piezoelectric materials, which convert motion-induced mechanical energy into electricity, show promise in actively accelerating the wound healing process. In this context, Wu et al. designed a self-aligned piezoelectric γ-glycine/PVA biofilm, optimized for use as a bioresorbable ultrasonic wireless electrotherapy device (b-WPUE) [58]. With concentric electrode devices spaced 1 mm apart, the system produced an electric field strength of approximately 220 mV mm^−1^, sufficient for effective wound electrotherapy. In a rodent model with artificial surgical wounds, the functionality of the b-WPUE was evaluated. Figure 9a illustrates the healing progress across four different treatment groups, where the wounds treated with b-WPUE and ultrasound exhibited significantly faster healing (~40% acceleration) compared to the control group. Notably the b-WPUE device alone did not alter the rate of healing, and while all wounds healed by day 15, the stimulated group achieved nearly complete recovery by day 10 (Figure 9b,c). No significant changes in weight were observed during treatment (Figure 9d). Histology analysis using H&E staining on tissue samples collected on days 6 and 12 further confirmed accelerated wound healing in the stimulated group (Figure 9e). These findings suggest that piezoelectric biofilms with excellent acoustic-electric conversion can be successfully integrated into b-WPUE device, offering an effective wireless electrotherapy approach that promotes wound healing rates in preclinical models.

#### 4.1.3. Nerve Regeneration

**Figure 10.**
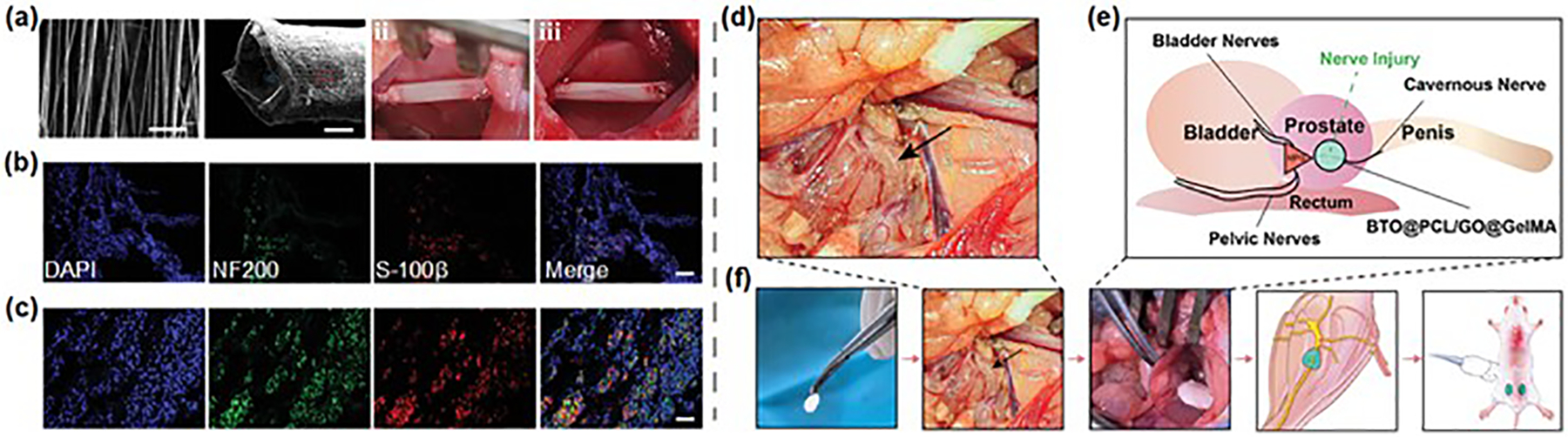
Piezoelectric conduits and nanopatches for the repair of peripheral nerve injuries. (**a**) SEM images of the aligned electrospun nanofibers and conduit; Photographs of implantation of (BPN US(−)) and (BPN US(+)). Scale bars are 5 μm in the first SEM image, 500 μm in the second SEM image. (**b**,**c**) Immunofluorescent staining of DAPI, NF200, and S-100*β* of (BPN US(−)) and (BPN US(+)) at eight weeks postoperatively. Scar bar is 20 μm. (**a**–**c**) Reproduced with permission [15]. Copyright 2024, Wiley. (**d**) Anatomical location diagram of cavernous nerve in rats. (**e**,**f**) Diagram of a band-aid-like nanopatch acting on neurologic erectile dysfunction rats. (**d**,**e**) Reproduced with permission [121]. Copyright 2024, Wiley.

Peripheral nerve injuries are common in clinical settings and often involve severe long-gap interruptions, significantly impacting patients’ daily lives and social activities [119,120]. For large-diameter peripheral nerve injuries (greater than 2 mm), researchers typically use biocompatible conduits to encase the damaged sciatic nerve, creating an artificial guiding channel to promote nerve regeneration. For example, Chai et al. developed a nerve guidance conduit composed of BTO nanoparticles-doped P(VDF-TrFE) aligned piezoelectric nanofibers, capable of repairing peripheral nerve injuries through ultrasound-triggered electrical stimulation [15]. These piezoelectric nanofibers achieved an open-circuit voltage of about 1.69 V under ultrasound stimulation at 0.75 W cm^−2^. To evaluate their clinical potential, conduits with varying treatments were implanted into 10 mm sciatic nerve defects in Sprague-Dawley (SD) rats (Figure 10a). Immunostaining of regenerated nerve tissue for S-100*β* (a Schwann cell marker) and NF200 (a neurofilament marker) confirmed nerve regeneration. Figure 10b,c demonstrate the presence of S-100*β*-positive cells and NF200-positive cells in the transversal nerves of experimental groups. Importantly, conduits subjected to ultrasound stimulation (BPN US(+)) exhibited higher expression of S-100*β* and NF200 compared to those without stimulation (BPN US(−)), demonstrating the enhanced nerve regeneration achieved through ultrasound-triggered wireless electrical stimulation. For smaller-diameter nerves (less than 1 mm) located in deeper anatomical sites and in contact with surrounding tissues, a nanopatch offers a promising alternative for nerve repair. Liu et al. introduced a band-aid-like nanopatch made of BTO@PCL/graphene oxide@gelatin methacryloyl for peripheral nerve repair using wireless ultrasound-electrical stimulation [121]. In a rat model of erectile dysfunction caused by peripheral nerve injury, the nanopatch adhered seamlessly to the damaged nerve, functioning like a band-aid (Figure 10d–f). This approach significantly improved corpus cavernosum nerve regeneration, enhancing tissue structure, erectile function, and a higher conception rate in female rats.

### 4.2. Drug Delivery

**Figure 11.**
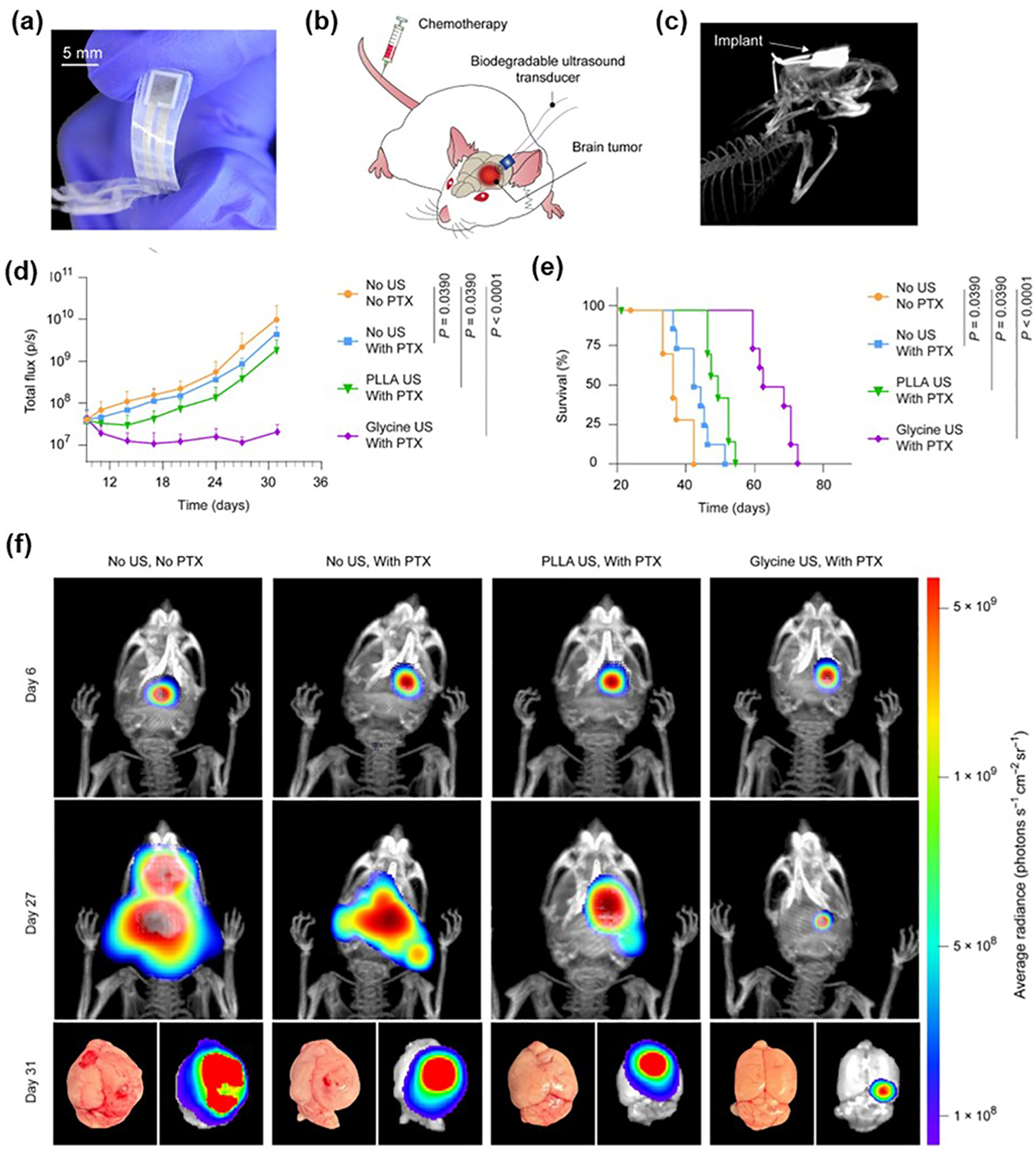
Implantable device made of glycine-PCL nanofiber membranes for facilitated delivery of chemotherapeutic drugs to brain tissue. (**a**) Optical photograph of a biodegradable glycine-PCL ultrasonic transducer. (**b**) Model of the glycine-PCL ultrasonic transducer in enhancing the delivery of chemotherapeutic drug to the brain for the treatment of GBM tumor. (**c**) Micro-CT image revealing the position of the implant in the animal. (**d**) Mean GBM tumor luminescence intensity levels of mice receiving different treatments. Data are means ± SD (n = 8, one-way ANOVA and Tukey multiple comparisons tests at day 31). (**e**) Kaplan-Meier survival of animals receiving different treatments (n = 8, log-rank test). (**f**) Bioluminescence images of GBM tumor growth in live animals and ex vivo images of GBM-bearing brains. Reproduced with permission [44]. Copyright 2023, The American Association for the Advancement of Science.

Precise drug delivery is essential for the treatment of various diseases, including cancer, tissue damage, and degenerative diseases. Recent research highlights the potential of piezoelectric materials in enhancing drug delivery systems by providing electrical stimulation, which can significantly improve the release and permeability of therapeutic molecules [122–124]. For instance, Lin et al. developed a controlled drug delivery system using piezocatalytic molybdenum disulfide nanoflowers as carriers for indomethacin, which can be activated by ultrasound to generate reactive oxygen species, facilitating targeted drug release for acute inflammation therapy [78]. In a paw edema model, a commercially available dressing functionalized with MoS_2_ containing indomethacin showed effective in vivo drug release, reducing paw swelling by up to 56% within 6 h. In another study, Nguyen et al. designed biodegradable, flexible piezoelectric glycine-PCL nanofiber membranes, which served as the core of an implantable ultrasonic transducer. This device, implanted into the brain, enhanced drug delivery across the blood-brain barrier (BBB), offering a novel approach for treating brain cancers [i.e., glioblastoma (GBM)] (Figure 11a) [44]. In an orthotopic U87MG-Luc GBM mouse model, the combination of paclitaxel (PTX) and ultrasound stimulation from the piezoelectric glycine-PCL transducer significantly improved anti-tumor efficacy (Figure 11b,c). Bioluminescence imaging revealed that mice treated with ultrasound-assisted PTX had the smallest tumors compared to other treatment groups (Figure 11d). Furthermore, the Kaplan-Meier survival analysis demonstrated that mice receiving the combined treatment had a prolonged median survival time of 65 days, with some surviving up to 72 days, which was significantly longer than those in other groups (Figure 11e). Furthermore, the corresponding images of the bioluminescence intensity of GBM cell at day 26, collected brain ex vivo imaging of the entire brain (Figure 11f) confirm that, in comparison to all other treatment groups, mice treated with glycine-PCL ultrasound-assisted PTX possessed the smallest tumor. All these findings indicate that biodegradable ultrasonic transducers based on piezoelectric glycine-PCL present a reliable and efficient option for future GBM treatments. These technologies have the potential to significantly advance biomedicine by overcoming the limitations of conventional external focused ultrasound, particularly in opening the BBB. Traditional methods face challenges such as significant attenuation of sound waves when passing through thick skulls and the difficulty of inducing effects repeatedly at precise locations. Piezoelectric systems offer a more targeted and reliable alternative.

### 4.3. Biosensors

**Figure 12.**
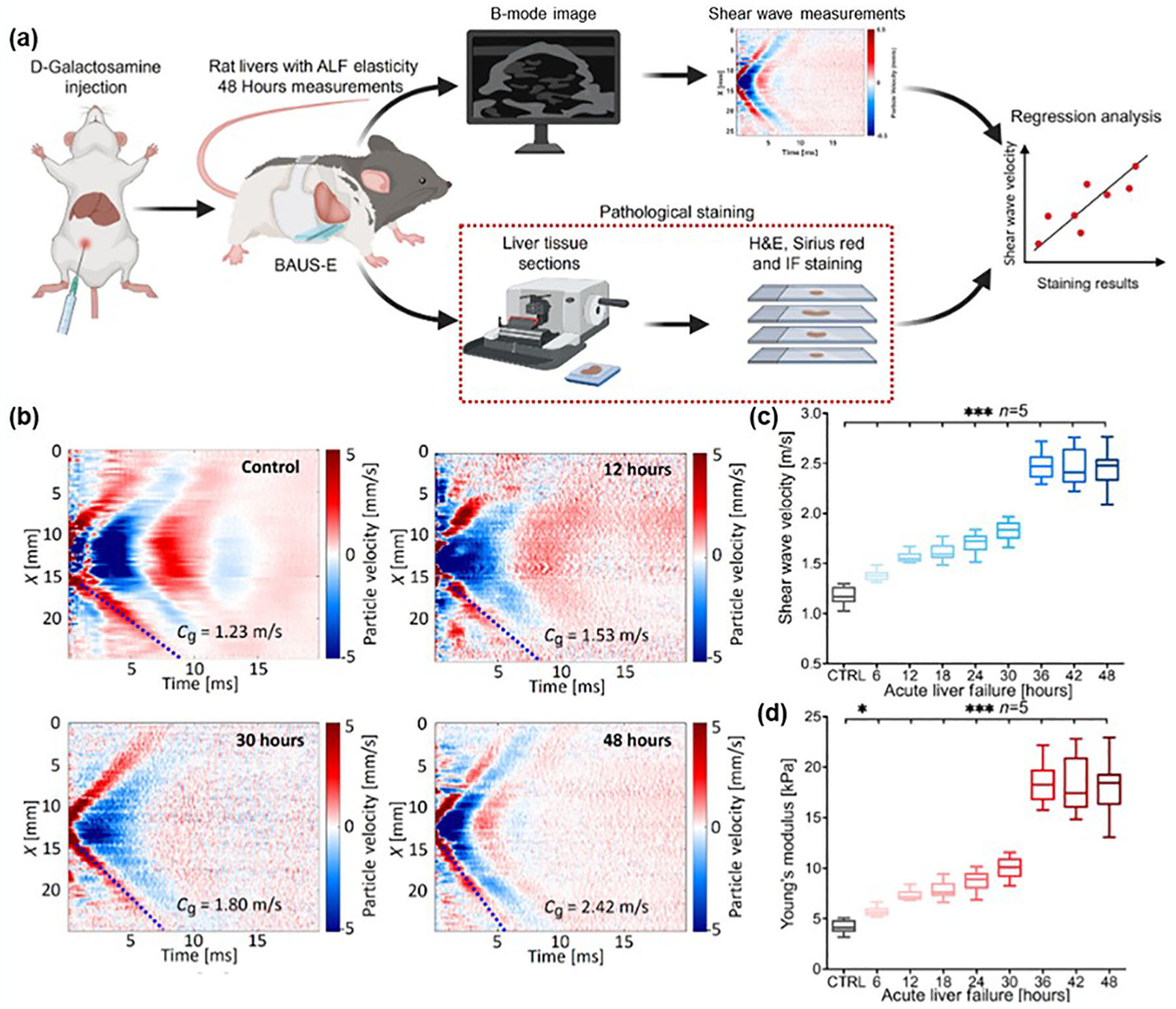
Wearable bio-adhesive ultrasound elastography based on a piezoelectric PZT layer for monitoring liver elasticity. (**a**) Schematic diagram of the procedure for assessing elasticity changes in rats with ALF. (**b**) Spatiotemporal maps of shear wave velocities. (**c**) Shear wave velocities were observed from 0 to 48 h using BAUS-E. (**d**) Trend of Young’s modulus changes in relation to the severity of rats with ALF over 48 h. IF, immunofluorescence. * *p* < 0.05, ** *p* ≤ 0.01, and *** *p* ≤ 0.001. Reproduced with permission [42]. Copyright 2024, The American Association for the Advancement of Science.

As digital healthcare technology continues to advance, wearable biosensors are being increasingly utilized to monitor multimodal physiological signals for various biomedical applications such as mental stress analysis, bladder volume monitoring, and blood pressure measurement [125–129]. A recent study introduced a wearable bio-adhesive ultrasound elastography (BAUS-E) device, featuring a piezoelectric lead zirconate titanate (PZT) layer. This device generated acoustic radiation force impulses to create shear waves, enabling the continuous evaluation of liver stiffness. Such measurements can serve as valuable biomarkers for tracking the progression of acute liver failure (ALF) through rapid, real-time changes in tissue elasticity. Figure 12a depicts the procedure for monitoring liver stiffness using a pharmacologically induced ALF animal model. Shear wave elastography (SWEs) data were collected over a 48-h period to evaluate the elasticity of rat livers using BAUS-E. The shear wave velocity (group velocity, Cg) values for the rat livers at four distinct time points—0, 12, 30, and 48 h—were recorded as 1.23, 1.53, 1.80, and 2.42 m/s, respectively, indicating a clear progression of liver injury (Figure 12b). A significant difference in liver stiffness was observed between normal livers and those affected by ALF at every time point (Figure 12c,d). Both BAUS-E and histological staining confirmed a strong positive correlation between liver stiffness and the severity of ALF. These findings suggest that BAUS-E could have extensive clinical applications, particularly for the non-invasive and continuous monitoring of internal organ changes in rapidly progressing conditions like ALF. The device’s potential for real-time tracking could be especially valuable in intensive care units and clinical settings where timely diagnosis and monitoring are critical.

In addition to these specialized applications, the use of piezoelectric materials in integrated diagnostic and treatment systems is propelling the development of innovative, non-invasive, and personalized medical technologies. These materials have been employed in efforts to achieve simultaneous visualization and treatment of malignant tissues. For instance, Shung et al. designed an integrated multifunctional confocal phased zirconate titanate array for noninvasive prostate tissue surgery, combining diagnosis and therapy capabilities [130]. The device features a triple row phased arrays: a 6-MHz array in the center row for imaging and two 4-MHz arrays in the outer rows for therapy. The 6-MHz imaging array consists of 128 elements with a 0.73λ = 188 μm pitch, 25 μm kerf, and 8 mm height, while each 4-MHz therapy array also has 128 elements with a 0.5λ = 188 μm pitch, 25 μm kerf, and 14 mm height. Both simulation and experimental results confirmed the device’s ability to provide real-time imaging during treatment. Similarly, Shrestha et al. developed a dual-mode piezo-composite transducer array integrating the therapeutic and diagnostic functions for image guided surgery [131,132]. This system offers immediate, spatially accurate lesion imaging and real-time feedback on tissue response to high-intensity focused ultrasound beams. As research advances, the application of piezoelectric materials in such integrated systems is expected to grow, enhancing the efficiency, accessibility, and effectiveness of healthcare delivery.

## 5. Conclusions and Outlook

Engineered piezoelectric metamaterials have emerged as an important innovation in biomedical applications, offering promising capabilities in addressing complex medical challenges and enhancing patient care. By integrating piezoelectric materials with metamaterial structures, researchers have developed highly specialized devices and systems that enhance both performance and functionality. The ability of these materials to efficiently convert mechanical stress into electrical energy has driven substantial progress in areas such as tissue regeneration, drug delivery, wound healing, and biosensing.

However, despite these advancements, a considerable gap remains between laboratory-scale demonstrations and practical clinical applications. As an emerging field, the development and deployment of piezoelectric metamaterials face several challenges. While many piezoelectric biomaterials exhibit excellent biocompatibility, their lack of biodegradability often requires secondary surgical procedures to remove them once they have served their purpose. To address this, there is increasing focus on developing naturally biodegradable piezoelectric materials. Modified proteins, for instance, are being explored as potential candidates to reduce the need for additional surgeries, thereby improving patient outcomes [89,133,134]. The degradation rates of biodegradable materials are often either too rapid or too slow for clinical needs. Many biomedical implants require functionality for several months to even years. Therefore, it is crucial to develop safe encapsulation materials or integrate degradation catalysts into piezoelectric devices to ensure they remain functional for the desired period and degrade predictably thereafter. For wearable biomedical devices, ensuring breathability and moisture management is crucial for user comfort and device reliability. Piezoelectric materials used in wearables must allow sufficient airflow and manage moisture effectively to prevent skin irritation [135–137]. Balancing piezoelectric functionality with user comfort is critical to the successful adoption of these devices in practical, long-term applications.

Moreover, the piezoelectric properties of materials can be significantly enhanced by subjecting them to high electric fields through polarization [71]. In their natural state, the dipoles within a material are randomly oriented, canceling each other out and resulting in minimal or no piezoelectric response. When an electric field greater than the saturation field but below the breakdown field is applied, the dipoles align, creating directional polarization [138]. Two commonly poling methods are direct current poling (DCP) and alternating current poling (ACP). DCP, a traditional technique, involves applying a DC voltage for a specific duration, allowing complete domain motion or switching during the poling process. In contrast, ACP is a dynamic approach that cyclically changes the polarization direction, offering significant potential for industrial applications. However, ACP does not allow sufficient time for internal stress relaxation, making it more difficult to achieve saturated poling compared to DCP [139,140]. To address this limitation, some researchers have combined the two methods, using ACP to change domain configuration initially, followed by DCP to ensure full domain reorientation [141]. Developing optimized polarization processes remains a key focus for advancing the fabrication of biological piezoelectric materials, paving the way for enhanced performance and broader applications. In summary, while piezoelectric metamaterials hold great promise in advancing biomedical technologies, overcoming these challenges will be essential for their widespread clinical adoption and sustained success.

## Figures and Tables

**Table 1.** Summary of piezoelectric properties of biomaterials.

	Piezoelectric Composition	Fabrication Method	Morphology	Piezoelectric Performance	Application	Refs.
0D	Amide-functionalized sulfonic acid bioorganic monomers	Hydrothermal method	Crystals	d_11_ = 15.9 pm V ^−1^	/	[76]
	Mn–Ti bimetallic organic framework	Hydrothermal method	Particles	d_33_ = 151 pm V ^−1^	Tumor therapy	[77]
	MoS_2_	Hydrothermal method	Nanoflowers	99 mV (measured by PFM under an applied voltage of 3 V)	Drug delivery	[78]
1D	Barium titanate (BTO)/PLLA	Electrospinning	Nanofibers	1.25 V (under the strain of 6 %)	Wound healing dressings	[79]
	BTO nanowires/polyvinylidene fluoride-trifluoroethylene P(VDF-TrFE)	Electrospinning	Coaxial nanofibers	18.2 V (under an impact force of 5 N)	Physiological multimodal sensing	[80]
	Al ion doped strontium titanate/titanium dioxide	Anodic oxidation, annealing, and hydrothermal reaction	Nanotubes	d 33 = 13.8 pm V^−1^	Antibacterial Therapy	[81]
2D	PVA/Glycine/PVA	Electric field-assisted water evaporation	Film	d_33_ = 6.6 pC N^−1^	Nanogenerator	[82]
	β-glycine/alginate/glycerol	Solvent-casting method	Film	d_33_ = 7.2 pC N^−1^	Sensor in artificial cochlea	[83]
	DL-alanine	Solution-Phase Self-Assembly	Biocrystal network	150 mV (under an impact force of 6 N)	Tactile sensor	[84]
3D	BTO/bioactive glasses	3D printing	Scaffold	d_33_ = 1.1 pC N^−1^	Bone regeneration	[85]
	BTO/P(VDF-TrFE)	Electrospinning and cross-linking polymerization with poly(N-isopropylacrylamide)	Nanofibrous hydrogel conduit	1.69 V (under US stimulation with 0.75 W cm^−2^)	Nerve regeneration	[15]
	1,3-propanediol, 2,3-butanediol, sebacic acid, succinic acid, and itaconic acid	Random copolymerization and hot press molding	Elastomer scaffold	0.9 V (50% horizontal elongation)	Repairing of skeletal muscles loss	[86]
